# An Information-Driven Approach for the Early Health Technology Sustainability Assessment and the Frugal Design of the Internet of Medical Things: Exploratory Study of Wearable Activity Monitoring Devices

**DOI:** 10.2196/88237

**Published:** 2026-07-31

**Authors:** Ernesto Quisbert-Trujillo, Nicolas Vuillerme

**Affiliations:** 1 Centre de Recherche en Santé Intégrée - CReSI, Univ. Grenoble Alpes, CNRS, Grenoble INP, LIG, Sangria, 6 chemin Saint Ferjus, Grenoble, 38700 La Tronche, France, 33 4 761 437 79; 2 Institut Universitaire de France, 75231 Paris, France

**Keywords:** health technology assessment, environmental sustainability, eco-design, frugality, Internet of Medical Things, wearables, step counting, physical activity, walking, information theory

## Abstract

**Background:**

Over the past decade, wearable activity monitoring (WAM) devices have become prevalent to improve quality of life and support the prevention and management of a wide range of health disorders. WAM devices based on the Internet of Medical Things (IoMT) paradigm provide a practical means of tracking physical activity, but their widespread adoption raises sustainability concerns. Meanwhile, health technology assessment and life cycle assessment are typically applied at advanced development stages, when empirical certainty about the final design and operating conditions is available, leaving little room for further improvements.

**Objective:**

This exploratory work provides the empirical foundations for an information-driven approach addressing this paradox in the early evaluation and conception of wrist-worn step counters, for which evidence suggests overdimensioned and unsustainable electronic designs. Specifically, we identify optimal resource-performance trade-offs in critical electronic components of frugal smartbands based on the data they collect and the information they preserve for step counting. This approach accounts for uncertainties in device reliability, users’ gait speeds, and material and energy consumption in final products.

**Methods:**

We conducted a secondary analysis of an accelerometer dataset characterizing wrist motion in healthy individuals walking at different speeds. The original x-, y-, and z-axis signals were progressively downsampled using cubic spline interpolation and discretized to quantify the information preserved, first across sampling frequencies and then with respect to changes in motion velocity. We also preliminarily assessed the viability of the downsampled signals for step detection by estimating percentage errors in peak and valley counts. Based on this, we constructed and evaluated 4 frugal smartband design archetypes, linking energy consumption and sampling frequency for 4 widely used accelerometers and combining essential electronic components, implementing a suboptimal asynchronous first-in-first-out (FIFO) algorithm at different sampling rates. Finally, we evaluated the environmental impact and circularity of these components through a streamlined analysis focused on raw materials.

**Results:**

About 70%‐90% of the information is lost in signals downsampled at very low frequencies (2‐5 Hz), whereas losses remain below 24% from 20 Hz onward. Substantial information loss also occurs when individuals walk briskly or jog (≥8 km/h), or walk below 8 km/h with sampling frequencies below 7 Hz or above 25 Hz. Step-counting accuracy is expected to be acceptable from approximately 11 Hz onward. Conversely, higher sampling rates rapidly saturate FIFO buffers and increase energy overhead, particularly when implemented in memory-dense components handling both processing and data transfer. Finally, gold and silver in transceivers and microcontrollers contribute substantially to resource depletion, while copper remains relevant for material recovery.

**Conclusions:**

These findings provide preliminary insights into the concurrent assessment and development of frugal WAM devices. This work extends the understanding of step detection under knowledge-constrained conditions and provides mechanisms to reduce uncertainty during early health technology assessment and eco-design.

## Introduction

Wearable activity monitoring (WAM) devices, such as smartwatches, fitness bands, and pedometers, are widely endorsed for promoting physical activity (PA) [1,2] and well-being [3]. Consequently, the WAM market is projected to grow significantly, from reported sales of US $71 billion in 2023 to more than US $186 billion by 2030 [4]. This rapid market expansion raises critical medium-term sustainability concerns. Under current trends, WAM devices are estimated to generate 3.4 Mt CO_2_-equivalent emissions by 2050, while also exacerbating challenges related to ecotoxicity, resource depletion, and e-waste management [5].

Yet, evidence also shows that the environmental impacts of wearables vary considerably. For instance, official sustainability reports for Samsung smartwatches reveal that resource depletion differs markedly across product families and models [6]. Moreover, the production of a midtier smartwatch accounts for approximately 5 kg CO_2_-eq of global warming potential [7], whereas this impact can increase by as much as 6-fold for a high-end model [8].

Different product families and premium devices undoubtedly offer a range of benefits. However, regarding PA monitoring performance, several works suggest that low-complexity devices are generally sufficient for approximate movement tracking [9,10], which appears adequate to encourage and sustain PA over the long term [11]. Furthermore, clinical studies have shown that accelerometer data collected over only a few days are sufficient to provide a reliable daily step count in people with neurodegenerative diseases [12]. Likewise, specific research has demonstrated that low data resolution does not significantly impair the detection of complex gait-related motor symptoms [13]. Taken together, this evidence suggests that limited resources and information can still be used effectively to monitor simple ambulation patterns.

From the perspective of national health authorities responsible for assessing and approving WAM devices, this insight is particularly important because a primary public health objective is to reduce disease morbidity through resource-efficient strategies that promote long-term PA. While health technology assessment (HTA) [14] and life cycle assessment (LCA) [15] are widely accepted methodologies for supporting this objective, their analytical reach is severely constrained during early product development due to limited empirical evidence [16]. Paradoxically, this stage also offers considerable freedom to make critical decisions that can positively influence not only the sustainability of final devices [17] but also their efficiency and ethical characteristics [18-20]. As a result, 2 related challenges emerge: an “eco-design paradox” [21,22], in which opportunities for environmental improvement decrease as product knowledge increases [23], and an early HTA paradox, in which the imbalance between uncertainty and evidence complicates decision-making in health care technology design and development [24].

In this exploratory work, we address these challenges by building on evidence suggesting (1) that low-complexity devices can effectively support approximate PA tracking [9-11] and (2) that limited information may still be sufficient to monitor simple ambulation patterns [12,13]. Specifically, this study investigates how the degradation of intrinsic information contained in accelerometer data influences the acquisition and use of knowledge and the management of uncertainty during the early-stage design and preliminary sustainability assessment of frugal WAM devices. To answer this research question, we analyze how sensor data availability affects the development and environmental evaluation of lightweight prototypes. In particular, we quantify the effects of incremental information loss on early design and on the energy consumption of basic algorithms operating under different motion velocities.

The aim of this exploratory research is therefore to examine the potential of a novel information-driven approach for addressing the difficulties and paradoxes associated with the early assessment and design of frugal WAM devices. In doing so, we contribute to the state of the art by providing knowledge relevant to the early design and preliminary environmental assessment of frugal archetypes, together with empirical evidence regarding the performance of final devices operating under specific and uncertain conditions. The remainder of this paper is organized as follows. The Methods section details our research approach applied to the specific case of smartbands, followed by the Results section presenting our preliminary findings. The Discussion section contextualizes the novelty and contributions of this work, addresses its limitations, and concludes with directions for future research.

## Methods

### Methodology Overview

Our research methodology consists of 3 successive steps: a secondary analysis of an existing accelerometer dataset, the construction of design archetypes for frugal smartbands, and a streamlined assessment of their environmental impact.

The first step is grounded in the principles of information theory and follows a data-information-knowledge process adapted for the ecological design and evaluation of Internet of Medical Things (IoMT) systems [25]. Its objective is to quantify how much information is preserved in signals describing the wrist movements of individuals walking at different speeds as the initial sampling rate is progressively reduced across the x-, y-, and z-axes. By doing so, we generate and use the knowledge required to manage critical uncertainties encountered during the early design and performance evaluation of archetypes, particularly those related to the movement speed of users and the energy consumption of final devices. We focus on energy consumption because it represents a major source of uncertainty in wearable design, as it is influenced by numerous stochastic factors, including usage context [26] and data acquisition, processing, and communication conditions [27].

The second step focuses on constructing electronic design archetypes for frugal smartbands based on the primary functions and capacities of their essential components, following the approach proposed by Quisbert-Trujillo and Morfouli [28]. Its objective is to support a worst-case analysis that addresses the uncertainty and limited knowledge regarding users’ walking speeds and the energy consumption of final devices. To achieve this, we evaluate the extent to which different design archetypes can support the acquisition, processing, storage, and transmission of raw accelerometer data under demanding operating conditions and at different sampling rates, independently of software-level optimizations.

The third step provides a high-level estimate of the environmental impact associated with the production of the essential components identified in the previous step and incorporated into the design archetypes. For conciseness and due to space limitations, this analysis was intentionally conceived as a lightweight environmental screening approach focused on the scarcity and potential recovery of raw materials. Its purpose is to provide rapid and pragmatic insights during early-stage product development and eco-design, where knowledge of final devices is typically limited. Specifically, the assessment is based on the identification and quantification of constituent refined materials using official manufacturer-declared material composition data. For simplicity, we focus exclusively on the contributions of gold, silver, and copper to abiotic resource depletion (according to the Environmental Footprint 3.1 impact assessment methodology and antimony-equivalent characterization factors expressed in kg Sb-eq). These materials were selected because of their documented prevalence in electronic devices [29-31] and their relevance to other environmental impact categories, including global warming [32].

**Figure 1.**
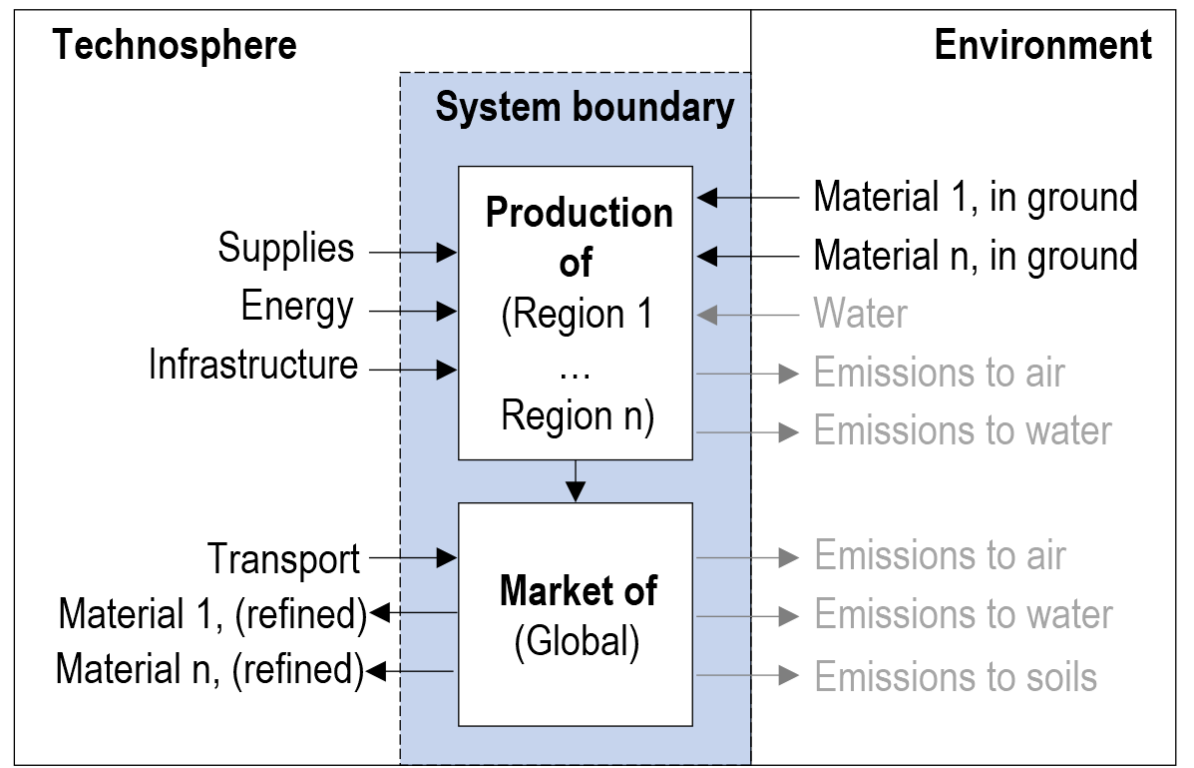
System boundary of the streamlined analysis conducted in this study. Only abiotic resource depletion (kg Sb-eq) was assessed; water consumption and emissions to air, water, and soil (shown in gray) were excluded from the system boundary.

Figure 1 illustrates the boundaries and limitations of this streamlined analysis, which accounts for all extractions involved in the global production and commercialization processes available in Ecoinvent 3.10 for the materials considered in this study.

In this sense, this streamlined analysis should not be interpreted as a comprehensive LCA study. Several standardized elements were intentionally excluded from the scope. Specifically, we did not include comprehensive life cycle inventory modeling of manufacturing processes of the electronic components of the design archetypes. Likewise, we did not model the technosphere and the environmental inputs and outputs associated with the use, transportation, maintenance, or waste treatment of final devices. In addition, no sensitivity analysis was conducted to support advanced interpretations of results.

Despite these limitations, the proposed streamlined approach provides a transparent, reproducible, and operationally practical method for integrating preliminary environmental considerations into early design-oriented analyses. As part of this step, we also conducted a conservative assessment of the recovery potential of gold, silver, and copper contained in the electronic components of the design archetypes. The objective was to establish a worst-case scenario for evaluating the minimum recyclability threshold of the final smartbands. This analysis is based on the respective material proportions and their corresponding functional recycling rates, according to Graedel et al [33].

### Secondary Analysis of the Accelerometer Data

The information theory concepts used in this part of the research methodology are marginal entropy, conditional entropy, and mutual information. Marginal entropy quantifies the uncertainty associated with a variable independently of another variable [34]. In contrast, conditional entropy quantifies the amount of uncertainty that remains about a variable when the value of another variable is known [35]. Mutual information quantifies the amount of information shared between two variables [34]. For two variables *X* and *Y*, the mutual information *MI*(*X*;*Y*) is mathematically defined as the difference between the marginal entropy of *X* (*H*(*X*)) and the conditional entropy of *X* given *Y* (*H*(*X*|*Y*)), as shown in Equation 1.

Equation 1: Mutual information formula


MI(X;Y)=H(X)-H(X|Y)

When the variables *X* and *Y* represent measurements of the same phenomenon, the mutual information *MI*(*X*;*Y*) can be interpreted as the amount of information about *X* that is also provided by *Y* [36]. This interpretation follows from the fact that *H*(*X*) quantifies the total uncertainty associated with the measurements represented by *X*, whereas *H*(*X*|*Y*) quantifies the uncertainty that remains after observing the measurements represented by *Y*.

In this work, *X* represents the original accelerometer data describing users’ movements at a reference sampling rate along the x-, y-, and z-axes, whereas *Y* represents the corresponding accelerometer data after downsampling. Therefore, if the downsampled signal *Y* preserves most of the measurements describing the relevant motion patterns of a specific PA, observing *Y* substantially reduces the uncertainty associated with the original signal *X* that describes this PA fully, resulting in a high value of *MI*(*X*;*Y*). Conversely, if the downsampled signal *Y* removes measurements that capture critical details of these motion patterns, the information provided by *Y* about the original signal becomes limited, and the mutual information decreases accordingly. In practical terms, this means that the downsampled signal no longer retains enough information from the original signal to adequately characterize the PA being monitored.

Following Kvålseth [37], we define the preserved information *PI*(*X*;*Y*) as the fraction of uncertainty in the original signal *X* that is resolved by observing the transformed signal *Y* or, equivalently, as the fraction of information contained in the original signal that remains after downsampling (Equation 2).

Equation 2: Preserved information formula


$$PI(X;Y)\text{=}\frac{MI\left(X;Y\right)}{H(X)}$$

So that


$$PI(X;Y)=\left\{ \begin{array}{l} \text{No information loss},PI(X;Y)=1,\\ \text{Partial preservation},0§lt;PI(X;Y)§lt;1,\\ \text{Complete information loss},PI(X;Y)=0. \end{array}\right.$$

### Data Preparation

The secondary analysis was conducted using a previously published dataset of treadmill walking tests performed by healthy participants at speeds ranging from 1.4 to 10 km/h [38]. In this exploratory study, only wrist accelerometer recordings collected with ActiGraph GT9X devices were analyzed, using the original triaxial signals sampled at 30 Hz as the reference data.

The original signals were progressively downsampled from 29 to 1 Hz using cubic spline interpolation. This approach was selected for its computational simplicity and suitability for benchmarking purposes, while remaining consistent with the Nyquist-Shannon theorem [39,40] given the frequency range associated with human walking [41]. In addition, previous studies have shown that spline interpolation can preserve the structural characteristics of a signal when generating lower-frequency representations from known data points [42].

After downsampling, both the original and downsampled signals were discretized using identical binning schemes and organized according to participant, walking speed, and signal axis. This procedure enabled direct comparisons between the reference and modified time series, as illustrated in Rashkovska et al [43]. Additional details regarding data preparation are provided in Multimedia Appendix 1.

### Preservation of Information

To quantify the preservation of information in the downsampled signals, the mutual information *MI*(*X*;*Y*) and the preserved information *PI*(*X*;*Y*) were computed using Equations 1 and 2 for each 1-second interval throughout the walking period. Mean values were then calculated for each signal axis and sampling frequency across all walking speeds. Subsequently, a deviation analysis was performed to evaluate the consistency of these results in accordance with conventional statistical methods [44].

In addition, changes in preserved information with respect to variations in walking speed and sampling frequency were quantified through *PI’*(*X*;*Y*) for each participant and signal axis, according to Equation 3.

Equation 3: Changes in the preserved information


$$PI^{`}\left(X;Y\right)\text{=}\left|\frac{PI{\left(X;Y\right)}_{s}\text{-}P{I(X;Y)}_{s`}}{P{I(X;Y)}_{f}\text{-}P{I(X;Y)}_{f`}}\right|$$

where


$$PI(X;Y{)}_{s}=PI\text{ for a given walking speed}$$

$$PI(X;Y{)}_{s^{\prime }}=PI\text{ for a walking speed lower than the given speed}$$

$$PI(X;Y{)}_{f}=PI\text{ for a given sampling frequency}$$

$$PI(X;Y{)}_{f^{\prime }}=PI\text{ for a sampling frequency lower than the given frequency}$$

More specifically, $PI^{\prime }\left(X;Y\right)$ is the derivative that systematically models the instantaneous rate of change of the preserved information variable. It quantifies how rapidly preserved information changes as a participant transitions from a given walking speed ($PI{\left(X;Y\right)}_{s}$) to a higher walking speed ($PI{\left(X;Y\right)}_{s`}$), while the accelerometer of the WMA device simultaneously transitions from a given sampling frequency ($PI{\left(X;Y\right)}_{f}$) to a lower sampling frequency ($PI{\left(X;Y\right)}_{f`}$).

### Evaluation of the Downsampled Signals

To estimate the viability of the downsampled signals for step detection, the procedure adopted in this exploratory study was divided into two stages and repeated across the x-, y-, and z-axes.

First, we identified and counted peaks and valleys in both the original and downsampled signals. This approach was selected because step-counting methods commonly rely on the detection of peaks and valleys in accelerometer data. Specifically, gray literature suggests that peak detection constitutes the basis of pedometer software implemented in widely used commercial sensors [45], while academic studies identify the detection of peaks [46] or peak-valley pairs [47] as standard approaches for step-counting algorithms.

In our study, peaks were identified using the find_peaks algorithm from the scipy.signal Python library. Detection was based on local extrema and constrained by a topographic prominence of 0.2 and a height threshold equal to the mean acceleration value plus half of its standard deviation for each axis. Valleys were identified using the same procedure applied to the inverted signals.

Second, we computed the percentage error of peak counts ($PE_{peaks_{i,j}}$) and valley counts ($PE_{valleys_{i,j}}$) for the downsampled signals at each walking speed *i* and sampling frequency *j*. The corresponding peak and valley counts obtained from the original signals were used as reference values, according to Equations 4 and 5.

Equation 4: Percentage error of peaks formula


$$PE_{{peaks}_{i,j}}=\left|\frac{PC_{i,j}-PC_{i,j}^{\prime }}{PC_{i,j}}\right|\times 100\%,\;\forall i\in \{1,\ldots ,29\},\;j\in \{1.4,\ldots ,10\}$$

Equation 5: Percentage error of valleys formula


$$PE_{{valleys}_{i,j}}=\left|\frac{VC_{i,j}-VC_{i,j}^{\prime }}{VC_{i,j}}\right|\times 100\%,\;\forall i\in \{1,\ldots ,29\},\;j\in \{1.4,\ldots ,10\}$$

where


$$PC_{i,j}=\text{Peak Counting}\in \text{the original signal for walking speed }i\wedge \text{ frequency }j$$

$$PC_{i,j}^{\prime }=\text{Peak Counting}\in \text{a downsampled signal for walking speed }i\wedge \text{ frequency }j$$

$$VC_{i,j}=\text{Valley Counting}\in \text{the original signal for walking speed }i\wedge \text{ frequency }j$$

$$VC_{i,j}^{\prime }=\text{Valley Counting}\in \text{a downsampled signal for walking speed }i\wedge \text{ frequency }j$$

In this manner, the results of this evaluation complement those of the previous section from a functional perspective. Specifically, they make it possible to assess the extent to which the information retained in the downsampled signals remains suitable for step-detection and step-counting applications.

### Ethical Considerations

This study did not involve any direct experimentation with human participants or patients. The analyses were conducted exclusively using secondary data derived from a previously published study. All data sources were appropriately cited, and data use complied with the conditions and guidelines specified in the original publication. The original study that generated the dataset used in our secondary analysis is part of a broader body of work executed under the European Union’s Horizon 2020 research and innovation program under the Marie Sklodowska-Curie grant agreement no. 823871 (iGame), and it was approved by the Ethics Committee of the Malaga Provincial Research of the Andalusian Health Service (RTC-iGAME 24092020). As reported by the authors of the original study, all participants accepted and signed the informed consent following the principles established in the Declaration of Helsinki.

## Results

### Preserved Information

Table 1 presents the mean preserved information values of the downsampled signals across all participants, grouped by sampling frequency.

**Table 1.** Preserved information in the downsampled signals across all participants, reported as mean percentages and standard deviations, for all walking speeds^a^.

Freq. (Hz)	Participant 1			Participant 2			Participant 3			Participant 4			Participant 5		
	X	Y	Z	X	Y	Z	X	Y	Z	X	Y	Z	X	Y	Z
1	4.22 (0.52)	4.23 (0.57)	4.20 (0.65)	4.25 (0.47)	4.23 (0.58)	4.19 (0.60)	4.22 (0.54)	4.19 (0.66)	4.20 (0.64)	4.23 (0.50)	4.21 (0.70)	4.19 (0.71)	4.22 (0.50)	4.23 (0.55)	4.20 (0.67)
2	8.45 (0.67)	8.39 (0.80)	8.34 (0.87)	8.44 (0.65)	8.39 (0.78)	8.36 (0.80)	8.44 (0.71)	8.31 (0.91)	8.35 (0.88)	8.45 (0.67)	8.28 (0.99)	8.30 (0.95)	8.44 (0.68)	8.39 (0.77)	8.36 (0.94)
3	12.55 (0.84)	12.51 (0.92)	12.40 (1.12)	12.64 (0.76)	12.50 (1.02)	12.49 (0.95)	12.58 (0.84)	12.41 (1.12)	12.42 (1.06)	12.63 (0.76)	12.38 (1.15)	12.40 (1.14)	12.64 (0.81)	12.58 (0.90)	12.45 (1.08)
4	16.72 (0.90)	16.62 (1.05)	16.53 (1.15)	16.76 (0.91)	16.62 (1.07)	16.56 (1.08)	16.71 (0.97)	16.48 (1.25)	16.52 (1.21)	16.74 (0.87)	16.41 (1.36)	16.43 (1.36)	16.76 (0.86)	16.61 (1.07)	16.54 (1.24)
5	20.84 (1.02)	20.68 (1.14)	20.60 (1.29)	20.79 (1.00)	20.72 (1.21)	20.64 (1.18)	20.76 (1.03)	20.56 (1.36)	20.57 (1.29)	20.85 (0.96)	20.46 (1.42)	20.45 (1.42)	20.85 (0.94)	20.70 (1.15)	20.47 (1.37)
6	24.87 (1.01)	24.74 (1.23)	24.54 (1.39)	24.93 (0.99)	24.75 (1.30)	24.71 (1.20)	24.86 (1.07)	24.56 (1.42)	24.65 (1.38)	24.92 (1.02)	24.41 (1.53)	24.46 (1.46)	24.92 (0.99)	24.75 (1.22)	24.58 (1.36)
7	28.87 (1.13)	28.78 (1.23)	28.58 (1.39)	28.98 (1.05)	28.76 (1.36)	28.74 (1.26)	28.89 (1.12)	28.50 (1.53)	28.60 (1.46)	28.89 (1.11)	28.47 (1.62)	28.43 (1.55)	28.89 (1.08)	28.69 (1.29)	28.46 (1.50)
8	32.92 (1.12)	32.76 (1.26)	32.52 (1.50)	32.94 (1.13)	32.73 (1.40)	32.66 (1.33)	32.86 (1.21)	32.48 (1.59)	32.51 (1.48)	32.93 (1.13)	32.37 (1.73)	32.30 (1.74)	32.97 (1.05)	32.74 (1.36)	32.53 (1.45)
9	36.80 (1.21)	36.65 (1.34)	36.42 (1.49)	36.86 (1.10)	36.65 (1.47)	36.66 (1.33)	36.85 (1.19)	36.40 (1.69)	36.44 (1.50)	36.90 (1.10)	36.29 (1.78)	36.22 (1.72)	36.91 (1.10)	36.69 (1.31)	36.42 (1.56)
10	40.78 (1.09)	40.52 (1.41)	40.35 (1.50)	40.71 (1.13)	40.55 (1.47)	40.51 (1.38)	40.66 (1.18)	40.33 (1.66)	40.30 (1.52)	40.76 (1.15)	40.15 (1.82)	40.11 (1.77)	40.81 (1.10)	40.56 (1.42)	40.23 (1.59)
11	44.63 (1.12)	44.38 (1.40)	44.14 (1.51)	44.66 (1.05)	44.38 (1.47)	44.35 (1.28)	44.56 (1.17)	44.08 (1.72)	44.23 (1.55)	44.61 (1.17)	43.97 (1.85)	43.96 (1.74)	44.64 (1.16)	44.41 (1.43)	44.06 (1.59)
12	48.47 (1.11)	48.20 (1.40)	47.96 (1.52)	48.50 (1.11)	48.18 (1.55)	48.21 (1.31)	48.39 (1.24)	47.94 (1.65)	48.00 (1.63)	48.46 (1.13)	47.77 (1.84)	47.74 (1.80)	48.50 (1.05)	48.21 (1.39)	47.95 (1.60)
13	52.19 (1.13)	51.98 (1.39)	51.67 (1.52)	52.29 (1.06)	51.99 (1.47)	51.96 (1.25)	52.15 (1.23)	51.65 (1.74)	51.73 (1.52)	52.21 (1.21)	51.61 (1.77)	51.48 (1.87)	52.23 (1.09)	52.01 (1.42)	51.60 (1.54)
14	55.92 (1.12)	55.74 (1.30)	55.35 (1.50)	56.00 (1.03)	55.67 (1.46)	55.70 (1.28)	55.92 (1.15)	55.37 (1.71)	55.49 (1.53)	55.95 (1.14)	55.35 (1.80)	55.18 (1.80)	55.96 (1.09)	55.68 (1.39)	55.37 (1.54)
15	59.60 (1.08)	59.33 (1.32)	59.05 (1.43)	59.66 (1.02)	59.31 (1.50)	59.32 (1.24)	59.57 (1.13)	58.97 (1.71)	59.07 (1.50)	59.67 (1.07)	59.00 (1.77)	58.80 (1.83)	59.63 (1.07)	59.41 (1.27)	59.04 (1.55)
16	63.23 (1.08)	63.01 (1.26)	62.71 (1.39)	63.29 (1.03)	62.91 (1.48)	62.95 (1.19)	63.18 (1.11)	62.64 (1.60)	62.73 (1.44)	63.22 (1.02)	62.53 (1.76)	62.50 (1.72)	63.29 (0.97)	63.06 (1.30)	62.66 (1.51)
17	66.81 (0.96)	66.56 (1.23)	66.25 (1.24)	66.76 (0.95)	66.53 (1.37)	66.52 (1.13)	66.69 (0.99)	66.22 (1.56)	66.25 (1.45)	66.74 (1.05)	66.12 (1.75)	66.06 (1.65)	66.81 (0.95)	66.58 (1.23)	66.27 (1.44)
18	70.24 (1.01)	70.04 (1.20)	69.84 (1.19)	70.28 (0.87)	70.04 (1.34)	70.02 (1.06)	70.22 (1.01)	69.73 (1.46)	69.78 (1.37)	70.28 (0.95)	69.62 (1.56)	69.58 (1.60)	68.18 (6.77)	70.05 (1.18)	69.71 (1.35)
19	73.61 (0.93)	73.45 (1.06)	73.22 (1.16)	73.72 (0.82)	73.35 (1.25)	73.41 (0.97)	73.60 (0.96)	73.16 (1.41)	73.25 (1.21)	73.68 (0.86)	73.06 (1.53)	73.00 (1.46)	73.69 (0.93)	73.45 (1.12)	73.14 (1.29)
20	76.95 (0.85)	76.77 (1.01)	76.52 (1.08)	76.98 (0.78)	76.74 (1.21)	76.71 (0.92)	76.89 (0.92)	76.56 (1.30)	76.58 (1.13)	76.98 (0.81)	76.42 (1.41)	76.32 (1.47)	77.02 (0.80)	76.74 (1.01)	76.47 (1.15)
21	80.15 (0.81)	79.98 (0.91)	79.84 (0.95)	80.19 (0.70)	79.99 (1.12)	80.04 (0.85)	80.15 (0.80)	79.76 (1.20)	79.81 (1.09)	80.20 (0.74)	79.69 (1.32)	79.64 (1.32)	80.21 (0.74)	80.09 (0.87)	79.78 (1.08)
22	83.31 (0.68)	83.15 (0.82)	82.96 (0.89)	83.33 (0.61)	83.11 (1.00)	83.14 (0.76)	83.27 (0.75)	82.96 (1.07)	83.00 (0.98)	83.33 (0.68)	82.82 (1.24)	82.84 (1.10)	83.34 (0.67)	83.18 (0.85)	82.95 (0.96)
23	86.32 (0.61)	86.19 (0.72)	86.03 (0.74)	86.38 (0.55)	86.18 (0.83)	86.18 (0.69)	86.28 (0.66)	85.99 (0.96)	86.07 (0.87)	86.31 (0.61)	85.91 (1.05)	85.89 (0.96)	86.32 (0.66)	86.23 (0.72)	85.98 (0.87)
24	89.19 (0.58)	89.05 (0.63)	88.95 (0.65)	89.19 (0.49)	89.04 (0.77)	89.08 (0.57)	89.14 (0.60)	88.92 (0.77)	88.95 (0.76)	89.19 (0.51)	88.83 (0.87)	88.78 (0.93)	89.23 (0.48)	89.09 (0.61)	88.96 (0.74)
25	91.88 (0.48)	91.79 (0.50)	91.70 (0.56)	91.90 (0.41)	91.79 (0.65)	91.80 (0.50)	91.86 (0.44)	91.69 (0.67)	91.71 (0.62)	91.87 (0.43)	91.61 (0.76)	91.56 (0.75)	91.90 (0.49)	91.80 (0.52)	91.66 (0.60)
26	94.39 (0.41)	94.33 (0.42)	94.24 (0.45)	94.41 (0.33)	94.33 (0.46)	94.33 (0.36)	94.36 (0.44)	94.25 (0.52)	94.25 (0.46)	94.39 (0.36)	94.20 (0.61)	94.19 (0.56)	94.40 (0.36)	94.34 (0.42)	94.22 (0.47)
27	96.65 (0.29)	96.61 (0.32)	96.57 (0.34)	96.66 (0.26)	96.60 (0.40)	96.61 (0.31)	96.62 (0.38)	96.54 (0.39)	96.56 (0.40)	96.65 (0.29)	96.51 (0.45)	96.51 (0.39)	96.65 (0.32)	96.63 (0.29)	96.56 (0.38)
28	98.58 (0.25)	98.55 (0.26)	98.55 (0.20)	98.58 (0.17)	98.54 (0.30)	98.56 (0.22)	98.57 (0.25)	98.53 (0.30)	98.53 (0.26)	98.58 (0.24)	98.52 (0.30)	98.50 (0.29)	98.59 (0.25)	98.56 (0.23)	98.54 (0.28)
29	99.98 (0.20)	99.98 (0.15)	99.98 (0.17)	99.97 (0.21)	99.99 (0.13)	99.97 (0.21)	99.97 (0.20)	99.98 (0.15)	99.98 (0.16)	99.98 (0.17)	99.97 (0.19)	99.98 (0.18)	99.97 (0.21)	99.98 (0.16)	99.96 (0.23)

a Full visualization and detailed results of the deviation analysis validating these tendencies are available in Multimedia Appendix 2.

The results reveal a consistent decrease in *PI*(*X*;*Y*) as the sampling frequency decreases. Specifically, the downsampled signals exhibit substantial information losses exceeding 91% at 2 Hz and 79% at 5 Hz. In contrast, information loss remains below 24% at 20 Hz, indicating a more moderate degradation of information.

### Changes in the Preserved Information

**Figure 2.**
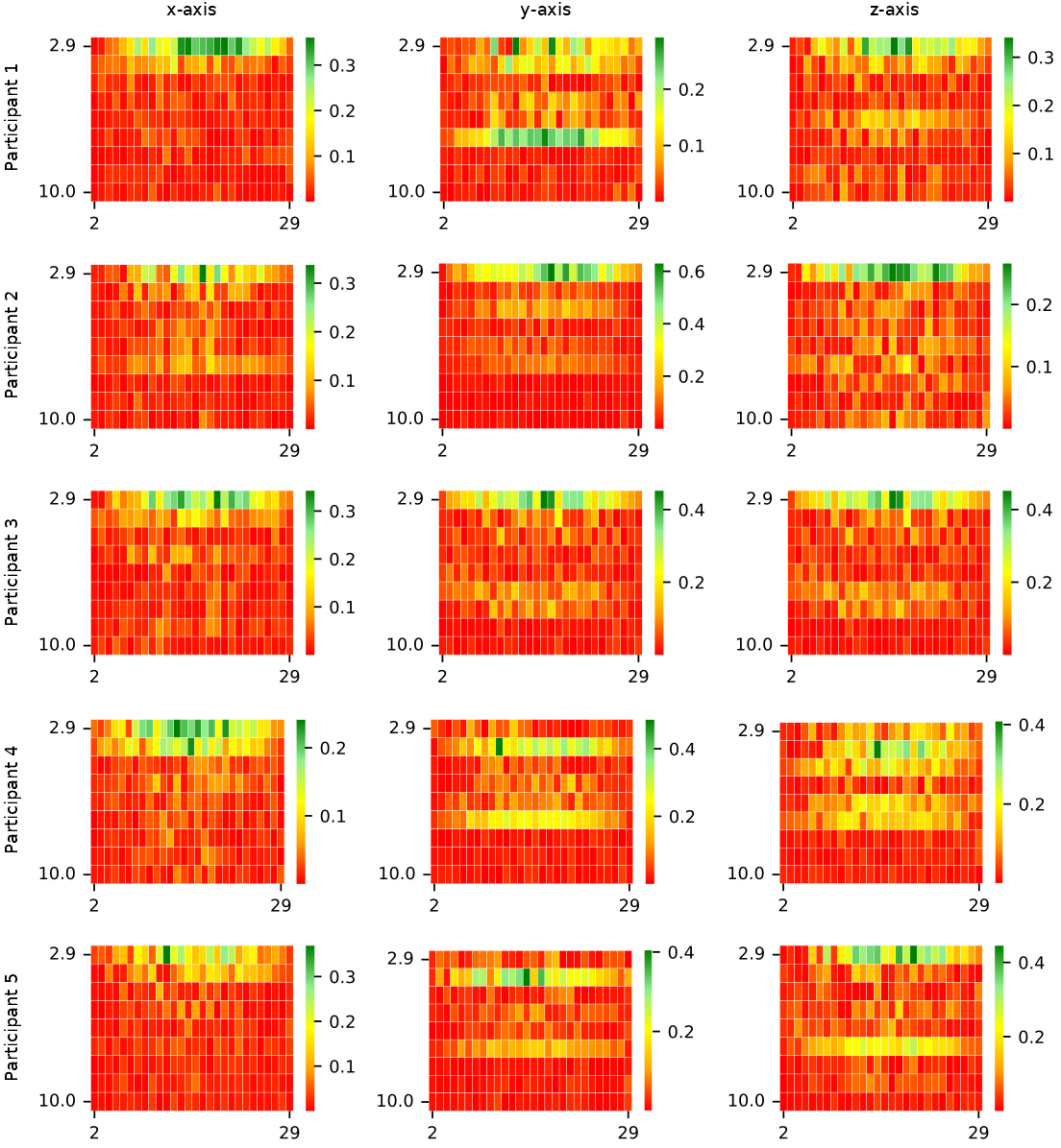
$PI^{\prime }\left(X;Y\right)$ values showing changes in preserved information as walking speed increases (from 1.4 to 2.9 km/h in the first row, from 2.9 to 4.3 km/h in the second row, and so forth) and sampling frequency decreases (from 30 to 29 Hz in the last column, from 29 to 28 Hz in the penultimate column, and so forth).

Figure 2 summarizes our preliminary findings regarding how preserved information varies with changes in walking speed and sampling frequency (*PI’*(*X*;*Y*)). Specifically, each cell within each heatmap represents the ratio between the changes in *PI*(*X*;*Y*) associated with a stepwise increase in walking speed and the corresponding changes in *PI*(*X*;*Y*) associated with a stepwise reduction in the accelerometer sampling frequency.

Across all participants and signal axes, substantial information losses are observed at both very low and very high sampling frequencies. However, no consistent pattern emerges when participants walk at speeds between 8 and 10 km/h.

In general, when participants walk at speeds between 1.4 and 2.9 km/h, information is preserved in all downsampled signals with only limited degradation at sampling frequencies between 7 and 25 Hz. However, this window progressively contracts as walking speed increases up to 6.5 km/h. For the y-axis, a noticeable increase in preserved information is observed for participants 1, 3, 4, and 5 when walking speed increases from 6.5 to 7.0 km/h. Although the cause of this behavior remains unclear, it does not alter the overall trends identified in this analysis.

### Results From the Evaluation of the Downsampled Signals

**Figure 3.**
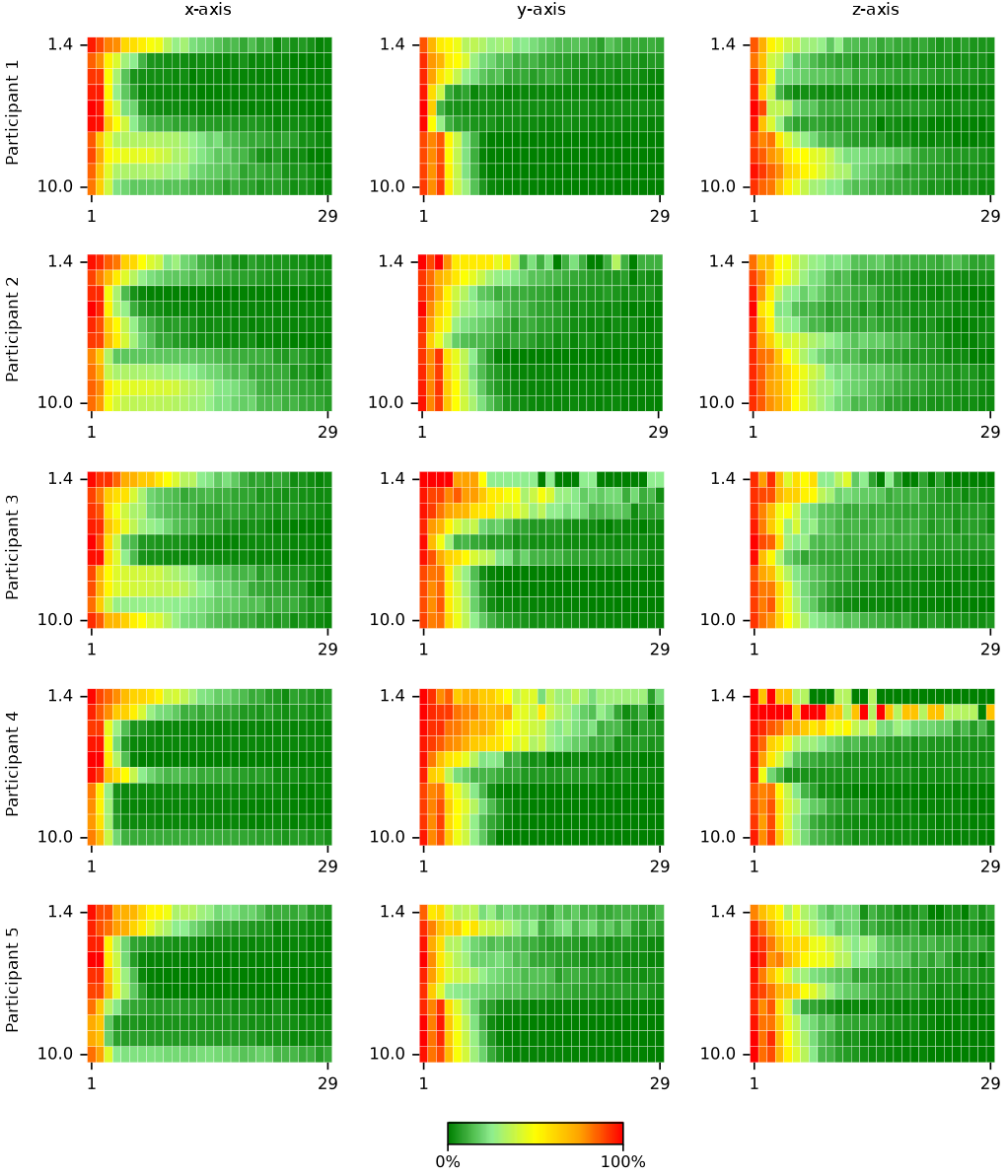
Percentage error (PE) of peak counts in the original and downsampled signals across all participants, sampling frequencies, and walking speeds. In each heatmap, gait speed (km/h) is shown on the vertical axis and sampling frequency (Hz) on the horizontal axis.

**Figure 4.**
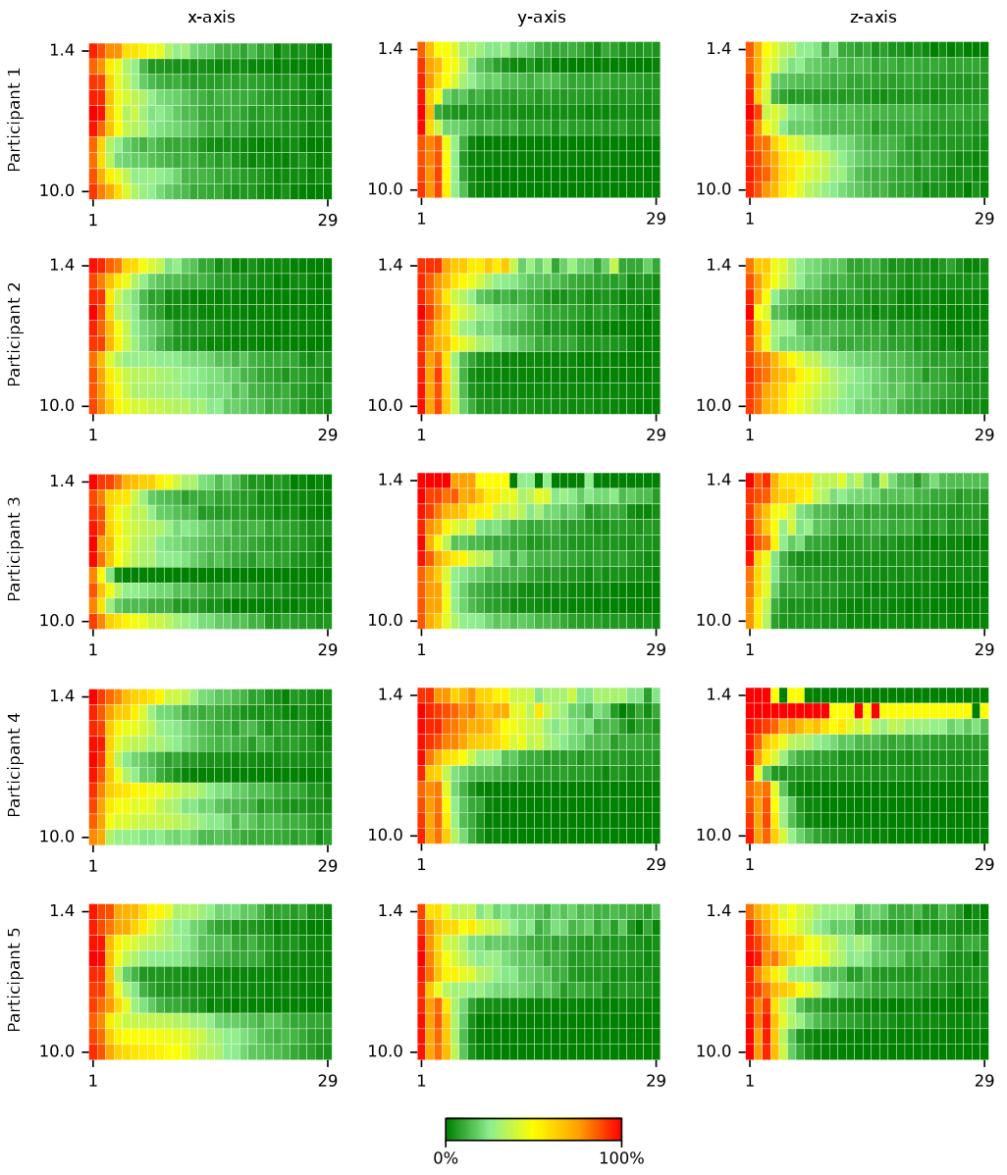
Percentage error (PE) of valley counts in the original and downsampled signals across all participants, sampling frequencies, and walking speeds. In each heatmap, gait speed (km/h) is shown on the vertical axis and sampling frequency (Hz) on the horizontal axis.

Figures 3 and 4 present our preliminary findings on the percentage error calculated from the peaks and valleys detected in the original and downsampled signals of all participants.

Across all participants and signal axes, the percentage error associated with peak and valley counts remains very high for downsampled signals sampled at very low frequencies (below 3 Hz). This result suggests that such signals are unlikely to support reliable step detection, regardless of walking speed.

In addition, the suitability of downsampled signals for step detection on the y-axis appears to deteriorate at walking speeds below 6 km/h when sampling frequencies fall below approximately 11 Hz. Under these conditions, the percentage error approaches 80%, suggesting a substantial reduction in detection performance.

### Design Delineation of Frugal Smartbands

The preliminary results presented in Figures 2-4 suggest that sampling frequencies around 15 Hz are likely sufficient to preserve the information for step detection during normal walking (ie, between 4.3 and 7 km/h). In addition, a broader frequency range (approximately 11‐25 Hz) may remain suitable when individuals walk at very slow speeds (below 3 km/h).

In practice, these relationships between sampling frequency, walking speed, and information preservation for effective step detection are often difficult to determine during the design of WAM devices. As a result, research and development efforts frequently rely on resource-intensive dynamic sampling strategies, including optimized duty-cycling approaches, on-chip processing, and advanced computing components. In this exploratory work, we use the findings from our secondary data analysis to examine how variations in sampling frequency and walking speed may influence the design of frugal smartbands. Specifically, we establish a set of hardware archetypes capable of supporting a suboptimal algorithm operating under different sampling rates. This approach allows us to define a worst-case scenario for energy consumption and user-behavior uncertainty, thereby providing insight into the robustness of the resulting devices.

**Figure 5.**
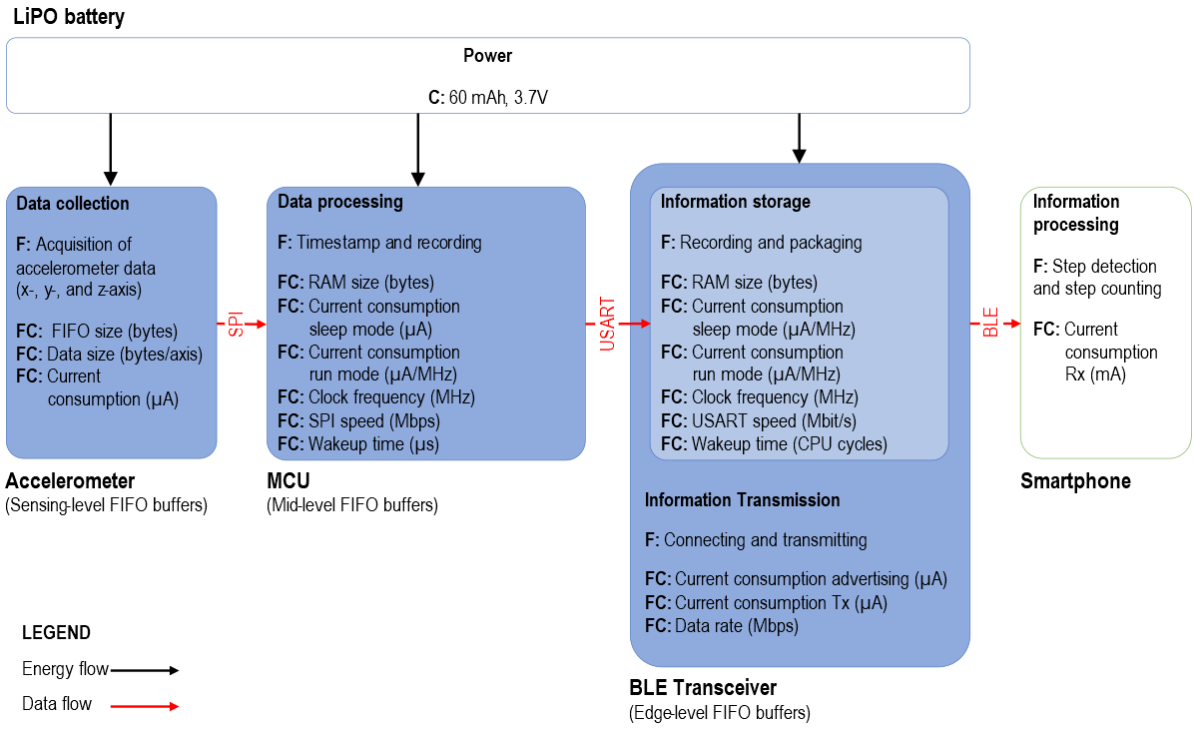
Framework for constructing design archetypes of frugal smartbands based on a simple asynchronous first-in-first-out (FIFO) strategy and a mapping of the capacities (C), functions (F), and function-capacities (FC) of their essential electronic components (adapted from the sustainable IoT design framework proposed by Quisbert-Trujillo and Morfouli [28]). BLE: Bluetooth Low Energy; MCU: microcontroller; SPI: serial peripheral interface; USART: universal synchronous/asynchronous receiver transmitter.

To support this analysis, we adopt a simple asynchronous first-in-first-out (FIFO) strategy as the basis for communication between 3 essential hardware components. We then relate the data acquisition, processing, and transmission functions of these components to their respective capacities to model and estimate energy consumption and battery autonomy across different design archetypes and sampling frequencies, according to the framework presented in Figure 5.

Each archetype consists of 3 core elements: an accelerometer, an ARM-based microcontroller (MCU), and a Bluetooth Low Energy (BLE) transceiver powered by a 60 mAh LiPo battery and organized as an asynchronous FIFO-based system. The accelerometer continuously acquires triaxial samples, which are transferred in bursts through the MCU and BLE transceiver before being transmitted to a smartphone. The energy model incorporates the current consumption and operating duration of each component in low-power, run, and sleep modes, including wake-up, processing, and data transmission functions (*F*).

Battery autonomy is estimated from the available battery capacity and the energy required to fill and transmit a complete BLE FIFO buffer, following the approach described by Tarkoma et al [48]. Because the FIFO filling time is determined by the accelerometer sampling frequency, this parameter directly influences the run and sleep durations of the remaining components and, consequently, the overall energy consumption of the system. Other factors affecting battery autonomy include communication throughput, processor clock frequency, workload, and FIFO/RAM capacities.

Using the component configurations and unfavorable operating conditions summarized in Tables 2 and 3, we evaluated all combinations of 4 accelerometers, 3 MCUs, and 3 BLE transceivers across multiple sampling frequencies. The objective was to identify the configurations capable of achieving at least 10 days of battery autonomy, a benchmark representative of current commercial wearable devices [49].

**Table 2.** Configuration settings for the accelerometers used to construct the archetypes^a^.

Capacities	MPU6000 [50]	LIS2DH12 [51]	LIS2DE12 [52]	LIS2DW12 [53]
FIFO^b^ size (bytes)	1024	192	192	192
Current consumption 1 Hz (μA)	—^c^	2	2	—
Current consumption 1.25 Hz (μA)	10	—	—	—
Current consumption 1.6 Hz (μA)	—	—	—	0.38
Current consumption 5 Hz (μA)	20	—	—	—
Current consumption 10 Hz (μA)	—	4	3	—
Current consumption 12.5 Hz (μA)	—	—	—	1
Current consumption 20 Hz (μA)	60	—	—	—
Current consumption 25 Hz (μA)	—	6	4	—
Clock frequency (MHz)	20	10	10	10
Voltage power supply (V)	3.46	3.6	3.6	3.6

a Official values as reported in the respective documentation cited in the references section.

b FIFO: first-in-first-out.

c Not available.

**Table 3.** Configuration settings for the MCUs^a^ and BLE^b^ transceivers used to construct the archetypes^c^.

	MCU			BLE transceiver		
Capacities	L011D3 [54]	L053C8 [55]	L073CB [56]	WB10CC [57]	WB50CG [58]	WB55CC [59]
RAM size (KBytes)	2	8	20	48	128	256
Current consumption sleep mode (μA)	0.54	0.8	0.86	28	41	41
Current consumption run mode (μA/MHz)	76	88	93	91	107	107
Current consumption advertising mode (μA)	—^d^	—	—	19.2	20	13
Current consumption transmission mode (μA)	—	—	—	8600	8800	5200
SPI^e^ throughput (Mbits/s)	16	16	16	—	—	—
USART^f^ throughput (Mbits/s)	—	—	—	4	4	4
BLE throughput (Mbits/s)	—	—	—	1	1	2
Wake-up time (μs)	5	5	5	—	—	—
Voltage power supply (V)	3.6	3.6	3.6	3.6	3.6	3.6

a MCU: microcontroller.

b BLE: Bluetooth Low Energy.

c Official values as reported in the respective documentation cited in the references section.

d Not available.

e SPI: serial peripheral interface.

f USART: universal synchronous/asynchronous receiver transmitter.

Based on these parameters, Tables 4-7 report the estimated runtime per full battery charge for different combinations. Archetypes capable of achieving at least 10 days of operational autonomy are highlighted in italics. The tables correspond respectively to the MPU6000, LIS2DH12, LIS2DE12, and LIS2DW12 accelerometers and include all of their reported sampling frequencies.

**Table 4.** Runtime per full charge of studied archetypes (in days) according to the low sampling rates reported for the MPU6000 accelerometer^a^.

	L011D3			L053C8			L073CB		
	1.25 Hz	5 Hz	20 Hz	1.25 Hz	5 Hz	20 Hz	1.25 Hz	5 Hz	20 Hz
WB10CC	*38.09*	*32.22*	*19.93*	10.78	10.25	8.57	3.18	3.13	2.96
WB50CG	*63.07*	*48.40*	*25.07*	*19.32*	*17.68*	*13.20*	6.45	6.26	5.59
WB55CC	*103.01*	*72.66*	*33.35*	*36.23*	*31.59*	*20.89*	*12.65*	*12.03*	*10.07*

a Archetypes capable of achieving at least 10 days of operational autonomy are highlighted in italics.

**Table 5.** Runtime per full charge of studied archetypes (in days) according to the low sampling rates reported for the LIS2DE12 accelerometer^a^.

	L011D3			L053C8			L073CB		
	1 Hz	10 Hz	25 Hz	1 Hz	10 Hz	25 Hz	1 Hz	10 Hz	25 Hz
WB10CC	*38.65*	*34.76*	*29.99*	9.57	9.31	8.93	3.21	3.18	3.14
WB50CG	*69.56*	*57.73*	*45.51*	*17.35*	*16.50*	*15.33*	6.58	6.46	6.27
WB55CC	*133.34*	*115.70*	*97.18*	*34.40*	*33.10*	*31.39*	*13.14*	*12.95*	*12.68*

a Archetypes capable of achieving at least 10 days of operational autonomy are highlighted in italics.

**Table 6.** Runtime per full charge of studied archetypes (in days) according to the low sampling rates reported for the LIS2DH12 accelerometer^a^.

	L011D3			L053C8			L073CB		
	1 Hz	10 Hz	25 Hz	1 Hz	10 Hz	25 Hz	1 Hz	10 Hz	25 Hz
WB10CC	*38.65*	*34.29*	*29.31*	9.57	9.28	8.87	3.21	3.18	3.13
WB50CG	*69.56*	*56.46*	*43.95*	*17.35*	*16.40*	*15.15*	6.58	6.44	6.24
WB55CC	*133.34*	*110.71*	*90.35*	*34.40*	*32.68*	*30.64*	*13.14*	*12.88*	*12.55*

a Archetypes capable of achieving at least 10 days of operational autonomy are highlighted in italics.

**Table 7.** Run time per full charge of studied archetypes (in days) according to the low sampling rates reported for the LIS2DW12 accelerometer^a^.

	L011D3		L053C8		L073CB	
	1.6 Hz	12.5 Hz	1.6 Hz	12.5 Hz	1.6 Hz	12.5 Hz
WB10CC	*39.35*	*34.86*	9.62	9.32	3.22	3.18
WB50CG	*71.85*	*57.96*	*17.49*	*16.52*	6.60	6.46
WB55CC	*144.52*	*123.85*	*35.10*	*33.73*	*13.24*	*13.05*

a Archetypes capable of achieving at least 10 days of operational autonomy are highlighted in italics.

Accordingly, and considering the capacities and parameters summarized in Tables 2 and 3, our preliminary results suggest that midlevel FIFO buffers should not be implemented across the full memory capacity of microcontrollers with moderate or relatively large storage capacities (such as the MCU L053C8). Under these conditions, transferring and writing large amounts of buffered data would prolong the active periods of other components, particularly BLE transceivers, thereby increasing energy overhead and potentially reducing battery autonomy below 10 days. This effect may become more pronounced at higher accelerometer sampling frequencies (above 20 Hz), which accelerates FIFO saturation throughout the system.

Conversely, this limitation could be mitigated when FIFO buffers are implemented across the full memory capacity of edge-level components, particularly the WB55CC BLE transceiver, provided that efficient radio communication capacities are available.

**Figure 6.**
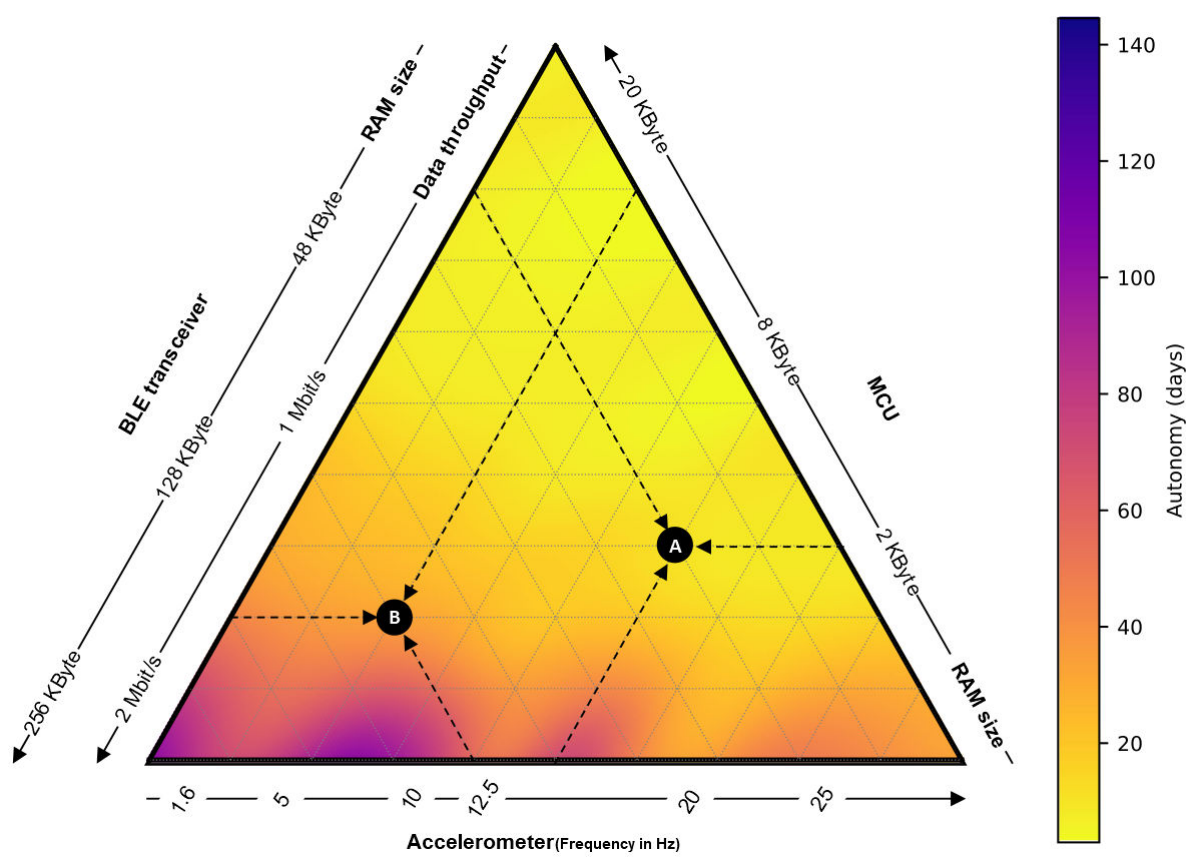
Practical design framework for frugal and efficient smartbands, according to the studied capacities of the electronic components that constitute the design archetypes. The scales on the right and left sides of the isosceles triangle show the RAM sizes of the studied microcontrollers (MCUs) and Bluetooth Low Energy (BLE) transceivers, as described in Table 3. The left scale also displays the data throughput of the BLE transceivers. The scale at the base shows the frequency range studied in our secondary data analysis, while the color scale on the rightmost side shows the battery autonomy range calculated from all combined function-capacities (FC) of the electronic components summarized in Figure 5, and the corresponding values and current consumptions reported in Tables 2 and 3. Each intersection within the triangle indicates the maximum battery autonomy (in days) associated with our worst-case use scenario, based on a suboptimal first-in-first-out (FIFO) strategy and on specific combinations of RAM capacity, communication throughput, and sampling frequency. For example, point A indicates that a battery autonomy below 20 days could be expected when combining a 2-Kbyte MCU RAM capacity (eg, provided by the L011D3 MCU) with a BLE transceiver offering less than 48 Kbytes of RAM and a data throughput below 1 Mbit/s (eg, the WB10CC transceiver) at an accelerometer sampling frequency of 15 Hz.

Figure 6 summarizes these findings in a practical framework intended to support the design of frugal yet robust smartbands for walking monitoring. The framework is based on the sensing, processing, storage, and transmission function-capacities (FC) of the electronic components that constitute the design archetypes evaluated in this study.

This preliminary framework helps visualize hardware trade-offs and the flexibility available for distributing computational workloads across system components. For example, point A suggests that designers should restrict MCUs to lightweight temporary storage functions and resource-constrained transceivers to simple data retransmission tasks. Such a configuration would support smartbands intended to operate primarily around 15 Hz while remaining close to a common commercial benchmark of approximately 10 days of battery autonomy.

In contrast, Point B indicates greater flexibility for implementing dynamic sampling strategies within the microprocessors of accelerometers, MCUs, or BLE transceivers. This configuration may be advantageous when supporting adaptive sampling frequencies below 12.5 Hz (but above 11 Hz), which correspond to situations in which users walk at very slow speeds. Under these conditions, a larger proportion of the available energy budget could potentially be allocated to onboard preprocessing or step-detection functions, as suggested by the estimated battery autonomy of 20‐40 days, even under the unfavorable operating conditions considered in this analysis.

### Environmental Impact Overview

**Figure 7.**
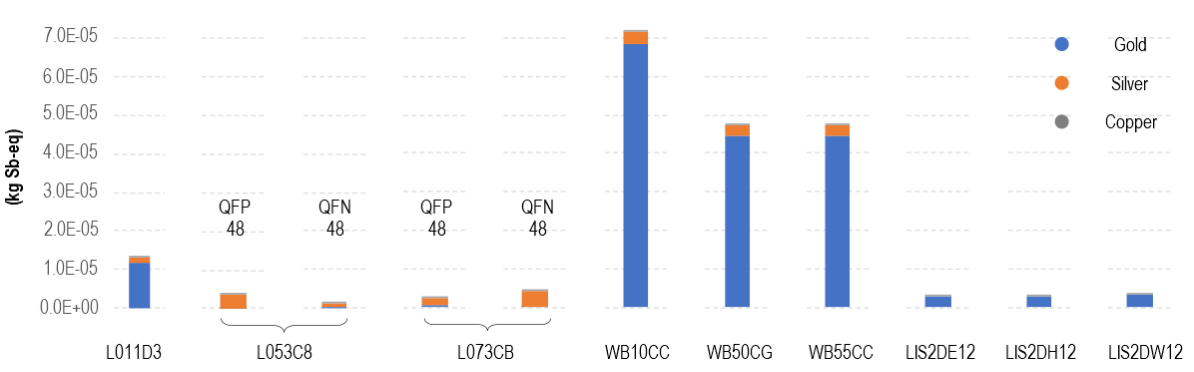
Preliminary results showing the resource depletion incurred in the production of the electronic components of the design archetypes, according to the Environmental Footprint 3.1 assessment method [60], expressed in kg Sb-eq.

Figure 7 presents the estimated resource depletion associated with the extraction of gold, silver, and copper required to manufacture the electronic components included in the design archetypes. Results for the MPU6000 accelerometer are unavailable because the manufacturer does not provide a material declaration.

According to our preliminary estimates, BLE transceivers contribute the most to resource scarcity, primarily because of the relatively large amount of gold used in their bonding wires. A similar pattern is observed for the L011D3 microcontroller and for all accelerometers included in this analysis. Silver also contributes noticeably to the impacts associated with BLE transceivers and with all variants of the L053C8 and L073CB microcontrollers.

**Figure 8.**
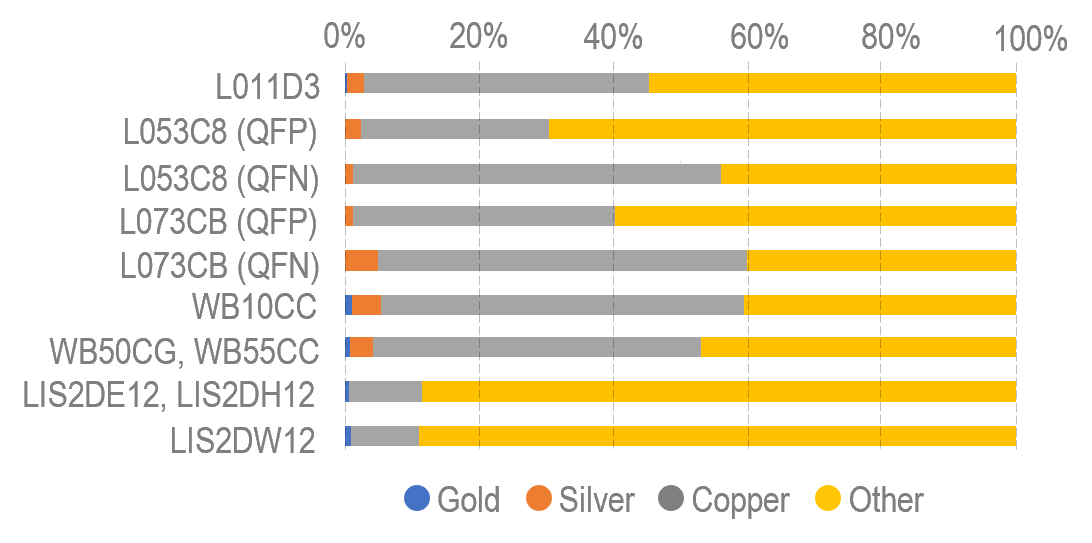
Proportions of gold, silver, and copper in the electronic components that comprise the design archetypes.

In contrast, copper contributes only marginally to the resource depletion results. However, its relatively high concentration in BLE transceivers and QFN-packaged microcontrollers suggests a considerable recovery potential under functional recycling scenarios, as illustrated in Figure 8.

Indeed, assuming a maximal functional recycling rate of 50% for gold, silver, and copper (as reported by Graedel [33]), substantial quantities of secondary copper could potentially be recovered and reintegrated into broader metallurgical production streams, as summarized in Table 8. Such material could also be reused in equivalent electronic applications following appropriate alloy adjustment processes, thereby establishing a defined lower bound for the recyclability potential of the resulting smartbands.

**Table 8.** Recovery yields of gold, silver, and copper in the studied electronic components that constitute the design archetypes (in mg).

Electronic component	Gold	Silver	Copper
L011D3	9.15E-02	6.67E-01	1.15E+01
L053C8 (QFP)	1.00E-03	2.21E+00	2.57E+01
L053C8 (QFN)	1.00E-03	5.69E-01	2.81E+01
L073CB (QFP)	6.50E-03	1.10E+00	3.65E+01
L073CB (QFN)	1.00E-03	2.53E+00	2.83E+01
WB10CC	5.27E-01	2.23E+00	2.76E+01
WB50CG, WB55CC	3.45E-01	1.70E+00	2.44E+01
LIS2DE12, LIS2DH12	2.20E-02	0.00E+00	4.34E-01
LIS2DW12	2.50E-02	0.00E+00	3.04E-01

However, realizing this recovery potential would depend on the effective implementation of closed-loop recycling strategies, as well as on the selective dismantling and segregated treatment of these components rather than their disposal within mixed electronic waste streams.

## Discussion

### Comparison With Prior Work

Various authors have successfully developed high-performing algorithms for wearables that maintain high accuracy even at very low sampling frequencies. For example, Straczkiewicz et al [61] developed a walking-recognition algorithm for smartwatches and wearable accelerometers capable of detecting and tracking activity periods with high sensitivity while operating at a sampling rate of 10 Hz. Likewise, other researchers have reported advanced step-counting algorithms achieving accuracies above 90% at sampling rates of at least 8 Hz, regardless of the device’s orientation [47].

The novelty of this exploratory work lies in moving beyond these algorithm-centered perspectives by focusing on the information content of the signals themselves. Specifically, we systematically quantify both the information preserved in downsampled accelerometer signals and their potential viability for step detection. By doing so, we provide designers and researchers with practical insights into how these two related aspects may influence not only the development of step-counting algorithms but also the early design and preliminary environmental assessment of frugal PA monitoring devices operating under baseline commercial constraints (ie, battery autonomy).

Our preliminary findings, based on the analysis of signals originally sampled at 30 Hz, reinforce the idea that triaxial accelerometer information remains useful for step detection during normal walking even at relatively low sampling frequencies (eg, below 15 Hz).

At the same time, our estimates suggest that signals sampled at very low frequencies (eg, below 3 Hz) retain limited information for reliable step detection and counting. Interestingly, our preliminary analysis also indicates that high frequencies (ie, above 26 Hz) may present limitations under certain conditions, particularly when individuals walk at low speeds, a tendency consistent with the observations of Fokkema et al [62]. Furthermore, when participants walk at very low speeds (2.9 or 4.3 km/h), information appears to be preserved and used more effectively within an intermediate frequency range between 11 and 25 Hz. Nevertheless, these observations should be interpreted with caution. Rather than generalizing the results across all signal axes, each axis should be considered separately, as evidenced by the percentage errors exceeding 80% for peak and valley detection along the y-axis.

Beyond signal analysis, the framework presented in Figure 6 extends previous research on medical-device design by providing an initial tool for delineating frugal smartband architectures during the early stages of product development, as recommended by Smith et al [63]. More specifically, it establishes a set of design archetypes that clarify how minimal hardware resources can be allocated according to different smartband usage requirements, consistent with the recommendations of Seva et al [64] regarding early Medical Device Readiness Levels.

From the perspective of environmental assessment within early HTA, our exploratory work offers an overview of several factors that influence the lifecycle of frugal devices. In particular, the environmental screening of the raw materials present in the electronic components that constitute the design archetypes identifies gold as a major contributor to resource depletion during production while simultaneously highlighting the potential importance of copper recovery during end-of-life management.

In addition, the framework presented in Figure 6 supports the initial formulation of preliminary operational scenarios for WAM devices based on component-level technical constraints. This capability helps reduce uncertainty regarding energy consumption, device performance, and user behavior during the early Technology Readiness Levels of new products, thereby addressing the well-recognized challenge of identifying pivotal parameters in early prototype assessments [24].

Overall, the proposed information-driven approach complements existing perspectives on the environmental assessment and design of wearable devices by providing both empirical quantification and practical decision-support tools. Specifically, the present work operationalizes the laboratory-scale assessment and use-scenario modeling activities described by Wentz et al [65] to estimate resource depletion and energy consumption at the prototype stage, thereby supporting the design of frugal smartbands through worst-case performance analyses. Similarly, this exploratory work provides an early-design instrument that facilitates the practical implementation of the broad recommendations synthesized by Gurova et al [66], particularly those related to energy efficiency and the allocation of computational and storage resources.

### Limitations

It is important to acknowledge the limitations of this exploratory study, as they directly affect the interpretation and generalizability of our preliminary findings.

First, our streamlined sustainability analysis of the electronic components included in the design archetypes was limited to a single environmental impact category. As a result, this analysis provides only a partial environmental perspective on WAM devices and should not be used in isolation as a substitute for a complete LCA or HTA.

Second, the secondary data analysis used to identify the “edge zones” associated with substantial information loss in accelerometer signals was based on a relatively small dataset comprising gait tests from only 5 healthy participants, each recorded for a maximum of 120 seconds. In addition, all data considered in this exploratory study were collected using a specific research-grade WAM device worn exclusively on the wrist. Consequently, both our preliminary findings and the resulting design recommendations should be interpreted with caution, particularly when considering applications beyond the specific population, device configuration, and experimental conditions examined in this study.

Third, several important dimensions typically considered within HTA were outside the scope of this work. These include broader socioeconomic aspects such as cost-effectiveness, robustness, and ease of repair. Accordingly, our objective was not to perform a complete HTA, but rather to provide a preliminary evaluation instrument for use during the early stages of device design.

Finally, caution is warranted when relating the results of our secondary data analysis to studies that compare accelerometer signals obtained from different devices. At present, no evidence supports the use of cubic spline interpolation to resample distinct signals prior to rigorous comparative analyses. Therefore, the findings reported here should not be interpreted as validation of such an approach in cross-device comparison studies.

### Conclusions and Perspectives

This exploratory study investigated how the degradation of intrinsic information contained in accelerometer data influences the acquisition and use of knowledge and the management of uncertainty during the early-stage design and preliminary sustainability assessment of frugal WAM devices. To answer this research question, we quantified both the preservation and practical utility of information contained in degraded accelerometer signals collected at different motion velocities. This knowledge was subsequently used to examine uncertainties related to user behavior and energy consumption during the early design of WAM device archetypes.

Overall, our preliminary findings suggest that both sampling frequency and gait speed play a critical role in determining how much information can be preserved and effectively used when individuals move at normal walking speeds. Based on the patterns identified in these variables, we observed the existence of “edge zones” in which information loss negatively influences the basic mechanisms used to detect gait-related PA (ie, peak and valley counting). These zones also influence hardware trade-offs, computational load allocation, and environmental screening activities performed during the early stages of device conception.

In this manner, this exploratory study provides initial tools and practical insights that may help address some of the challenges and paradoxes that complicate HTA and LCA during the early design and development of WAM devices. Through our secondary data analysis, we extend current knowledge regarding the minimum data requirements that researchers and designers should consider when developing gait-recognition solutions under different sampling conditions.

Furthermore, the application of the design framework presented in Figure 6 provides preliminary evidence that austere hardware architectures combined with limited data inputs may still achieve acceptable operational performance, offering a potential alternative to unnecessarily complex electronic designs. Finally, the integrative framework and environmental screening proposed in this work may assist HTA practitioners and regulatory bodies in developing an initial understanding of some factors contributing to the ecological burden of WAM devices, particularly those associated with raw materials during production and end-of-life stages within a usage-oriented context.

Nevertheless, these tools and insights should be interpreted and applied cautiously. The findings of this exploratory study remain constrained by two main factors: (1) the limited scope of the environmental overview, which focused exclusively on a single environmental impact category, and (2) the relatively small dataset used in the secondary data analysis.

More broadly, this work illustrates the application of part I of our previous methodology for the sustainability assessment and development of IoMT [25]. In the present study, this methodology was applied to the specific context of wearable devices, with particular emphasis on how uncertainty and information influence the early assessment and sustainable design of smartband prototypes. The applicability of our preliminary findings to final products will be examined in part II of the methodology, which will be detailed in a forthcoming study. This future work will benefit from richer proprietary datasets generated through our ongoing investigations of unsupervised mobility, involving the assessment of average daily steps in large cohorts of participants with motor impairments (ie, more than 100 individuals) monitored through consumer-grade wearables under free-living conditions [67,68]. These datasets will enable a more robust evaluation of the preliminary observations derived from the secondary data analysis presented here. Likewise, the clinical outcomes and socioeconomic implications of frugal devices derived from the proposed design archetypes remain important topics for future investigation.

Finally, extending this line of research to other domains, such as medical imaging, represents a promising opportunity to further investigate how information influences both clinical accuracy and the environmental impact of medical devices. This is particularly relevant in fields where these outcomes depend directly on the quantity and quality of the underlying data.

Through these future investigations, we aim to gather additional evidence to examine one of the central hypotheses underpinning our broader research program that “information is the message from which a sustainable digital-based health service or paradigm can exist, and with which specific barriers for integral assessment and eco design can be surpassed” [25].

## Supplementary material

10.2196/88237 Multimedia Appendix 1 Detailed description of the data preparation process for the secondary analysis.

10.2196/88237 Multimedia Appendix 2 Spread and variability of the preserved information across all speeds.

## Abbreviations

BLE: Bluetooth Low Energy
FIFO: first-in-first-out
HTA: health technology assessment
IoMT: Internet of Medical Things
LCA: Life Cycle Assessment
MCU: microcontroller
PA: physical activity
WAM: wearable activity monitoring
