# Supplementary material for: An Information-Driven Approach for the Early Health Technology Sustainability Assessment and the Frugal Design of the Internet of Medical Things: Exploratory Study of Wearable Activity Monitoring Devices
Source: JMIR Mhealth Uhealth. 2026 Jul 31;14:e88237. doi: 10.2196/88237 (PMC13427070; doi:10.2196/88237)
Supplement: Multimedia Appendix 1 [file mhealth-v14-e88237-s001.docx]

# **Multimedia Appendix 1.** Detailed description of the data preparation process for the secondary analysis

Our secondary analysis is based on a previously published dataset comprising walking tests performed by healthy participants at different speeds (1.4, 2.9, 4.3, 5, 6, 6.5, 7, 8, 9, and 10 km/h). To build this dataset, participants wore two WAM research-grade devices (Actigraph GT9X) on the wrist and on the thigh, and walked on a treadmill for 120 seconds. For practical reasons, in this exploratory work we used only accelerometer data generated from the wrist movements and assumed the frequency used by the authors (i.e., 30Hz) as the original signals for movements in the x-, y- and z-axes.

To obtain the modified signals for each participant and walking speed test, we used cubic spline interpolation to downsample the original signals stepwise from 29 Hz to 1 Hz. We employed this method for benchmarking convenience and computational simplicity, as the modified signals originate from the same original recordings and comply with the Nyquist-Shannon theorem. Indeed, given that the natural frequency of human walking on a treadmill (1.5-2.4 Hz) lies well below the Nyquist frequency of the original 30 Hz signal (15 Hz), this compliance is inherently satisfied. Under these conditions, prior evidence in other disciplines has demonstrated that spline interpolation preserves the structural integrity of the original signal across all downsampled signals generated from its known data points.

While data from the full two-minute trials were analyzed for participants 2 and 4, 118-second windows were used for participants 1, 3, and 5 to account for missing data during the first and last seconds of the recordings. The original and modified signals were discretized and organized by participant, walking speed and axis. Equal-size bins were applied to both signals. The range for computing the number of bins was determined based on the minimum and maximum values for the x, y, and z axes observed in the dataset. Additionally, equal-size bins of 3600 and 3540 units (for participants 2 and 4, and 1, 3 and 5, respectively) were applied to both the reference and interpolated time series to allow a comparative analysis of the downsampled and original signals, resulting in a temporal resolution of 1/30 second per bin. Figure S1 below illustrates some outcomes of this step, showing the discretization processes for the original signals of wrist movement for participants 2 and 4, and for the corresponding modified signals interpolated to two different frequency rates.


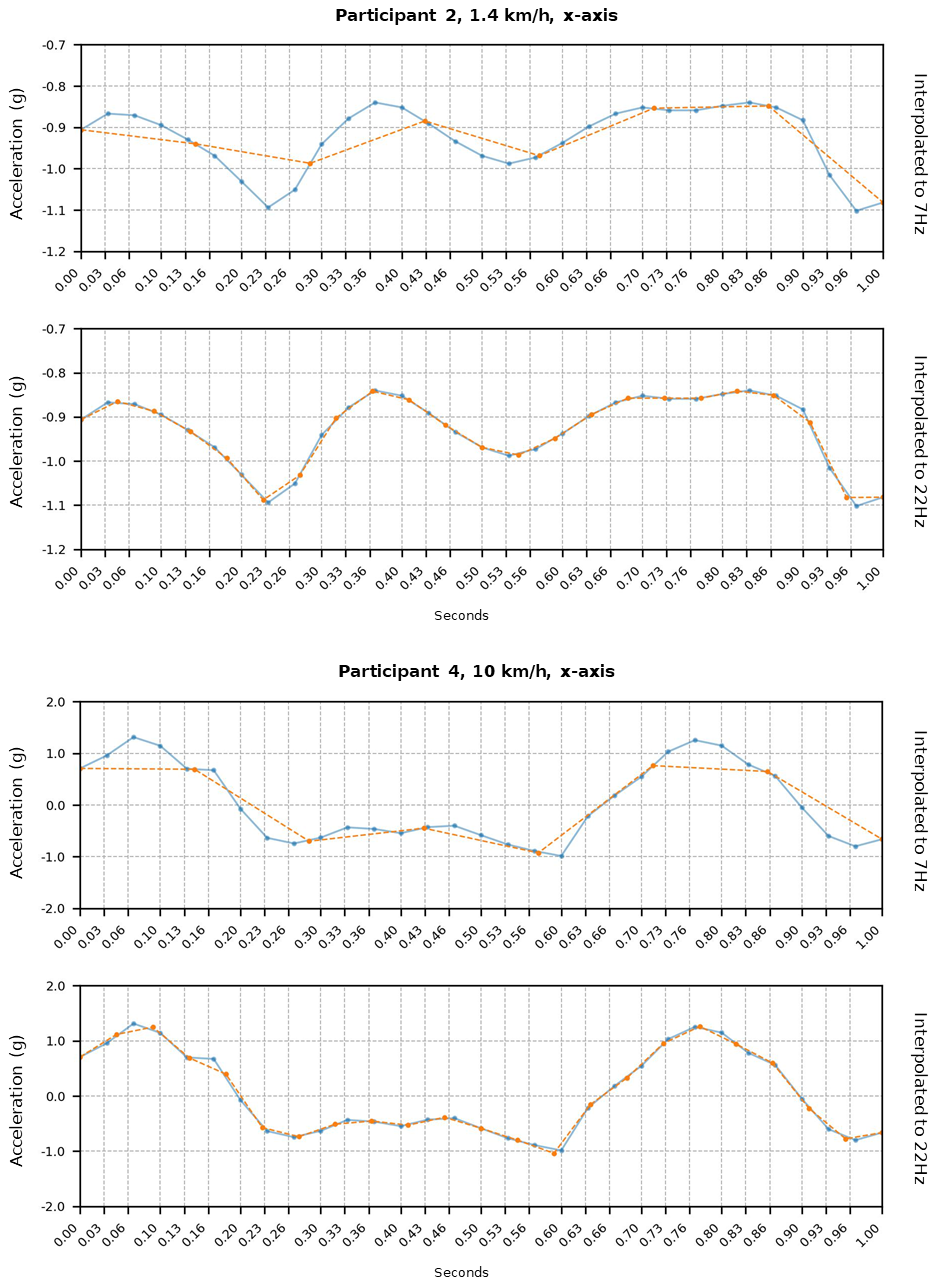
**Figure S1.** A portion of the original signals (blue) and interpolated signals (orange) from the wrist movements of participants 2 and 4, walking at the lowest and highest motion velocities over one second (here showing for the x-axis). During the discretization process, the number of bins for the accelerometer data is variable (shown here as five bins for participant 2 and four bins for participant 4 for illustrative purposes), while the number of bins for the time series is fixed at 3600 and 3540 bins (showing only 30 bins due to space limitations). The Mutual Information is computed for each second of walking, based on the amount of information carried in the original signals that is accessible when observing the modified signals, for each bin corresponding to the accelerometer data and the time series.
