# Supplementary material for: An Information-Driven Approach for the Early Health Technology Sustainability Assessment and the Frugal Design of the Internet of Medical Things: Exploratory Study of Wearable Activity Monitoring Devices
Source: JMIR Mhealth Uhealth. 2026 Jul 31;14:e88237. doi: 10.2196/88237 (PMC13427070; doi:10.2196/88237)
Supplement: Multimedia Appendix 2 [file mhealth-v14-e88237-s002.docx]

# Multimedia Appendix 2. Spread and variability of the preserved information across all speeds


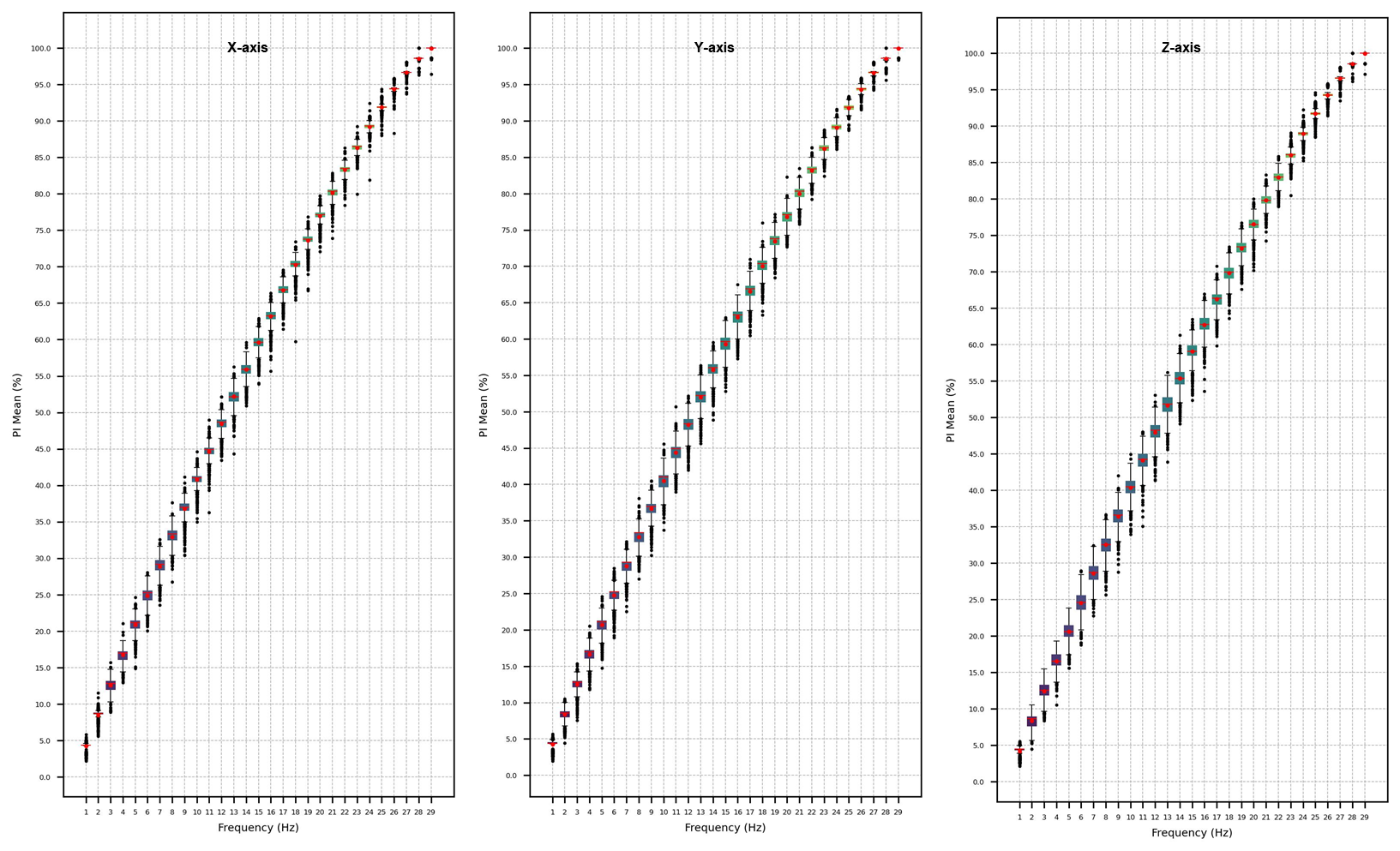
**Figure S1.** Means (red points), median (red lines), spread and variability of the preserved information across all speeds (by studied frequencies). Participant 1.


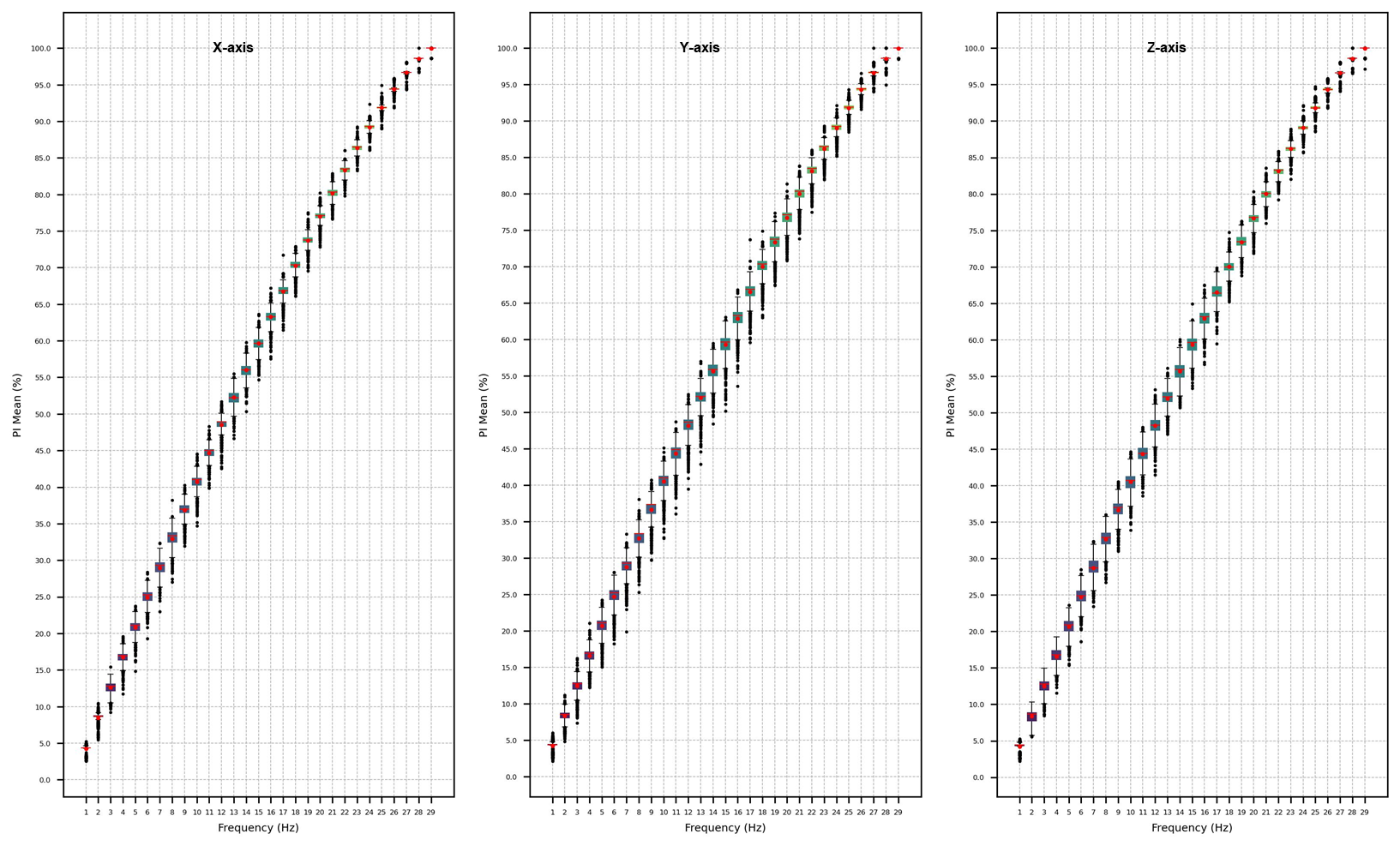
**Figure S2.** Means (red points), median (red lines), spread and variability of the preserved information across all speeds (by studied frequencies). Participant 2.

**Figure S3.** Means (red points), median (red lines), spread and variability of the preserved information across all speeds (by studied frequencies). Participant 3.


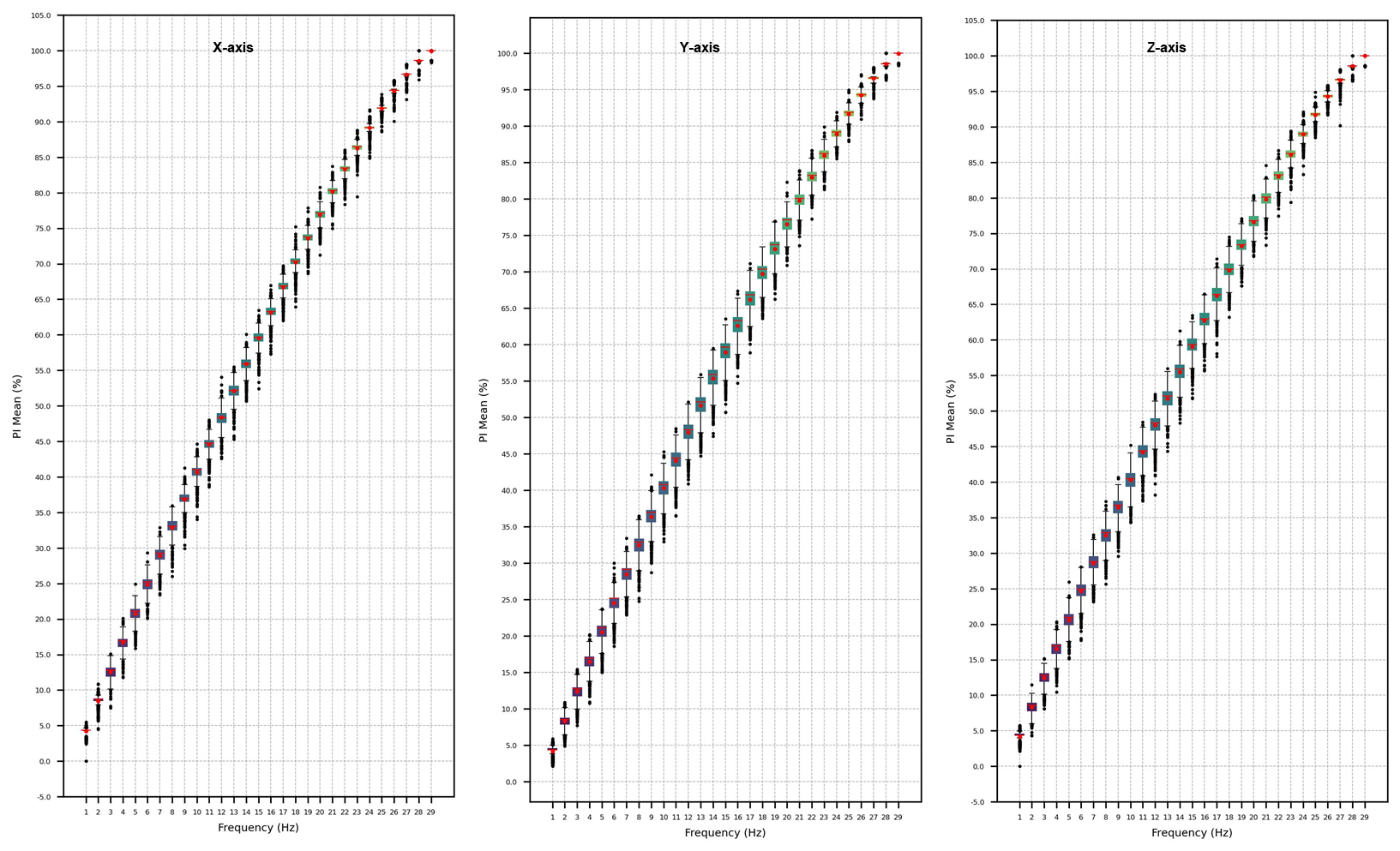


**Figure S4.** Means (red points), median (red lines), spread and variability of the preserved information across all speeds (by studied frequencies). Participant 4.


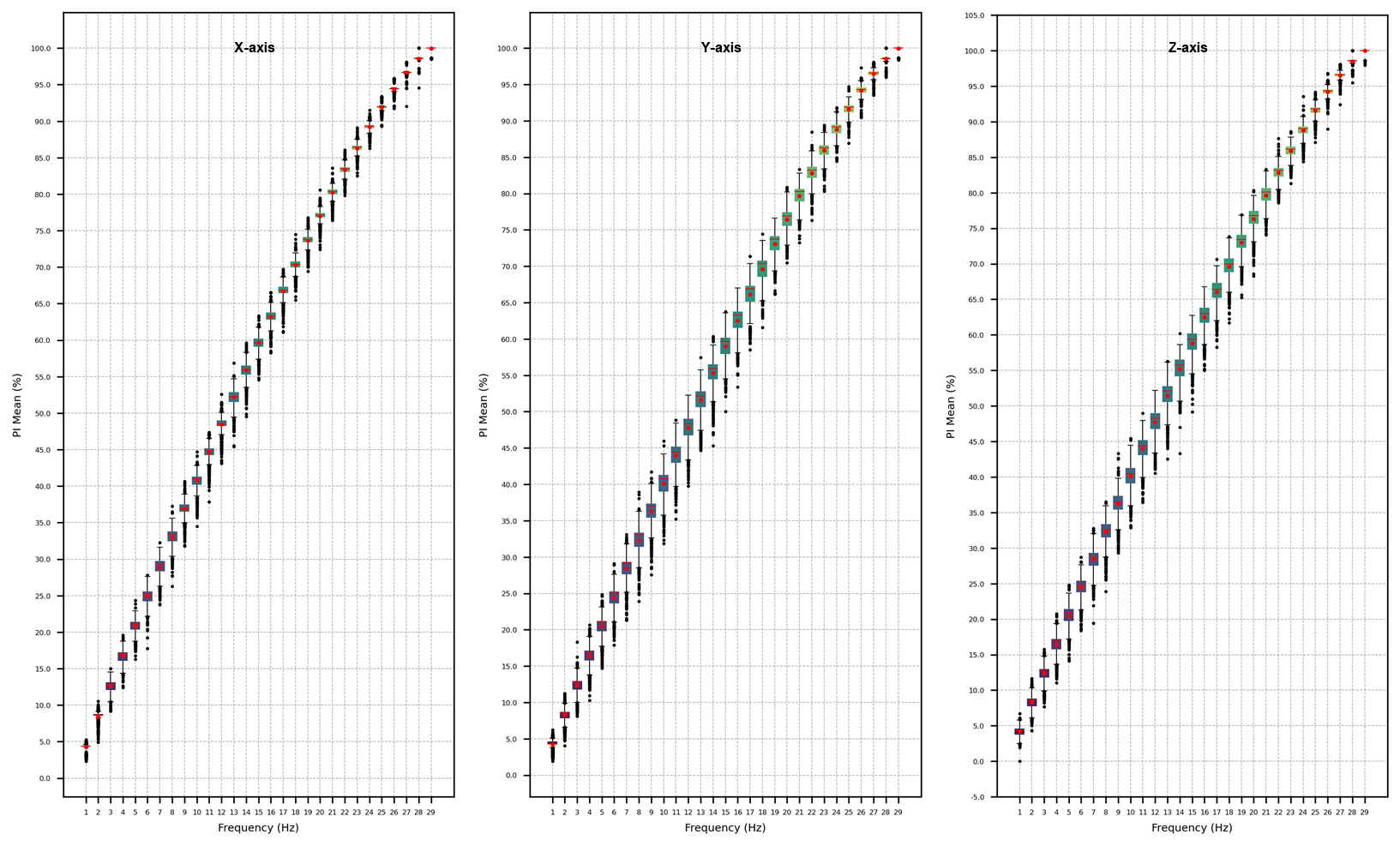


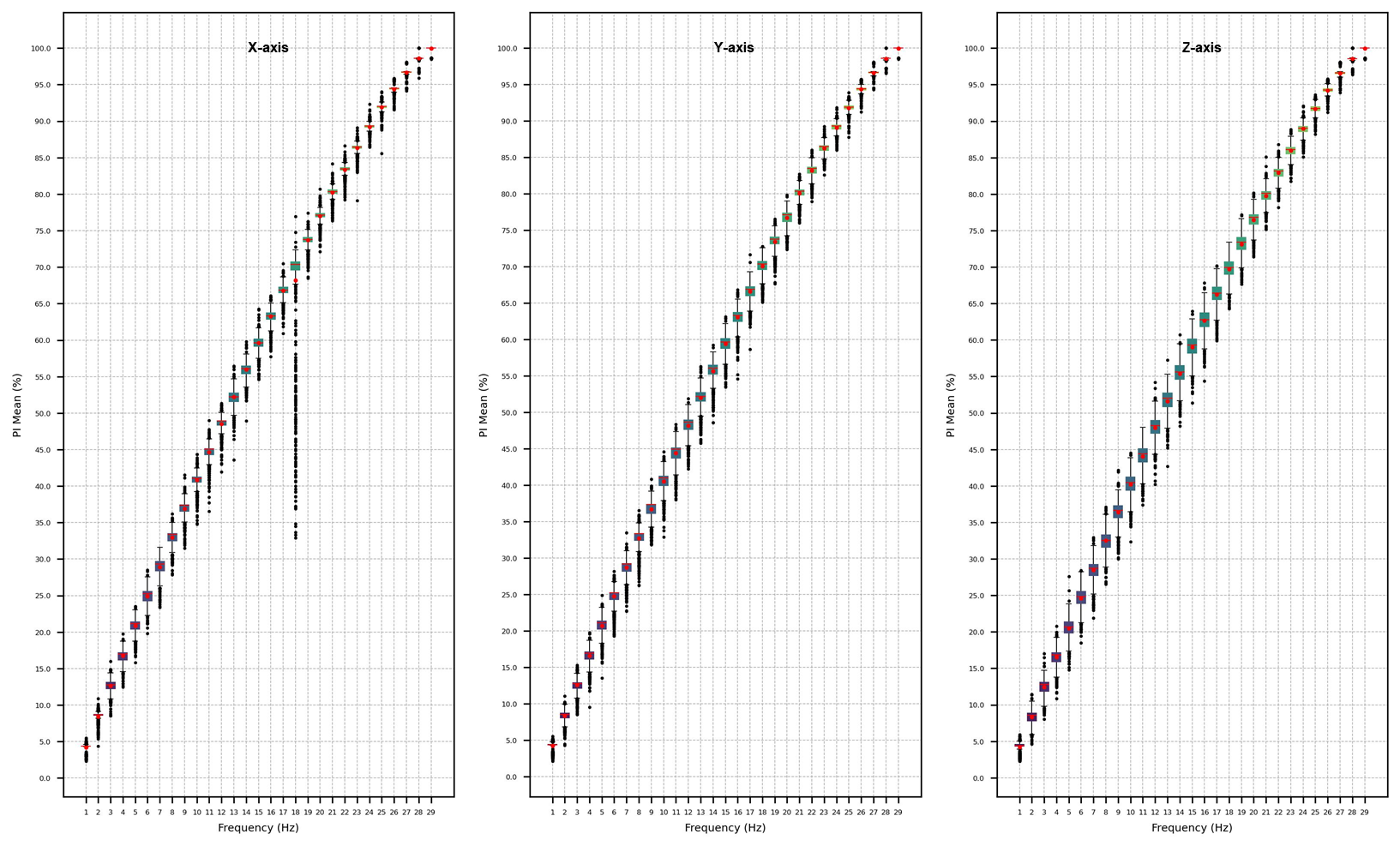
**Figure S5.** Means (red points), median (red lines), spread and variability of the preserved information across all speeds (by studied frequencies). Participant 5.
